# GB20-5A8-31, an anti-TL1A antibody for treating inflammatory bowel disease

**DOI:** 10.3389/fimmu.2026.1682346

**Published:** 2026-02-03

**Authors:** Yu Huang, Qiongying You, Tianqi Yao, Yanrong Tong, Xiaodong Yang, Xiangling Zhang, Xiaoting Huang, Qin Chen, Feng Lu, Huiming Li, Junfeng Wang, Suofu Qin

**Affiliations:** 1 Drug Discovery, Center for Research and Development, Kexing Biopharm Co., Ltd., Shenzhen, Guangdong, China; 2 High Magnetic Field Laboratory, Hefei Institutes of Physical Science, Chinese Academy of Sciences, Hefei, Anhui, China

**Keywords:** anti-TL1A, drug discovery, IBD, inflammation, therapeutics antibody

## Abstract

Inflammatory bowel disease (IBD) remains a critical unmet medical challenge, with a substantial number of patients experiencing ineffective treatment or therapeutic failure over time. Here, GB20-5A8-31, a novel anti-TNF-like ligand 1A (TL1A) antibody, was engineered for high potency and superior developability. GB20-5A8–31 exhibited ultra-high affinity (KD = 5.11×10–^11^ M) for human TL1A, potent inhibition of TL1A signaling *in vitro*. Meanwhile, GB20-5A8–31 demonstrated potent anti-inflammatory effects and anti-fibrotic tendencies in both the 2,4,6-trinitro-benzenesulfonic acid-induced rat and dextran sulfate-induced hTLIA-transgenic mouse acute IBD models. Its favorable pharmacokinetic profile, including an extended half-life (T_1/2_ = 248.54 h) in hFcRn-transgenic Sprague-Dawley rats, supports sustained target engagement. Crucially, GB20-5A8–31 exhibits advantageous biophysical properties—including high stability, solubility, and low aggregation—which facilitate the development of high-concentration formulations aimed at improving patient compliance. In summary, these findings indicate that GB20-5A8–31 is a therapeutic candidate for IBD with promising preclinical efficacy and the potential to advance to the Chemistry, Manufacturing and Controls research.

## Introduction

Inflammatory bowel disease (IBD), which includes Crohn’s disease (CD), ulcerative colitis (UC), and unclassified IBD, is a chronic intestinal inflammation mediated by dysregulated immune responses (1). The common symptoms associated with IBD include abdominal pain, diarrhea, bloody stools, and weight loss, with severe cases potentially leading to systemic complications (2). The persistent nature of the inflammation associated with IBD has been demonstrated to increase the risk of gastrointestinal cancers and other extra-intestinal malignancies, significantly affecting patients’ health.

The etiology of IBD is multifactorial, involving genetic predisposition, environmental triggers, dysregulated immune system, and alterations in the gut microbiota (3, 4). While current IBD therapeutic agents, such as anti-tumor necrosis factor (TNF) antibodies, integrin inhibitors, and interleukin (IL)-12/23 antagonists, can improve outcomes of some patients, notable limitations remain (1): Pathway singularity: These therapies primarily block a single inflammatory mediator (TNF-α, or IL-12/23) without modulating multiple overlapping pathogenic pathways, limiting their effectiveness in dealing with the complex mechanism of the IBD. Approximately 30-40% of patients do not respond to current biological therapy, and nearly half of initial responders lose efficacy within the first year of treatment (5–7) (2). Lack of fibrosis intervention: Although these therapies increasingly control inflammation, they do little to prevent the progression from intestinal inflammation to fibrosis. About 30-50% of IBD patients eventually progress to intestinal fibrotic stenosis, often leading to surgical resection (8–10) (3). Safety issues: As first-line biologics for treating IBD, anti-TNF antibodies significantly increase the risk of infections and malignant tumors (11, 12). These bottlenecks underscore the imperative of developing multi-pathway interventions and precision-targeted therapeutic strategies.

Tumor necrosis factor-like cytokine 1A (TL1A), encoded by the *TNFSF15* gene, is a member of the tumor necrosis factor superfamily (TNFSF) and functions by binding to its receptor, death-domain receptor 3 (DR3) (13). Recent research has revealed that TL1A plays a crucial role in the pathogenesis of IBD. Genetic polymorphisms in *TNFSF15* are associated with both susceptibility and severity of IBD (14). TL1A expression is significantly elevated in intestinal tissue and systemic circulation of patients with IBD compared to healthy individuals (15, 16).

As a powerful upstream regulator of the intestinal inflammatory pathway, TL1A exerts multifaceted effects on the intestinal inflammation observed in IBD. It amplifies the immune response by activating the NK-κB signaling pathway in naïve or memory T cells and promoting their differentiation into major effector T cell lineages implicated in IBD, including T helper cell (Th)1, Th2, Th9 and Th17 (17). TL1A amplifies Th cell responses by synergizing with pro-inflammatory signals, which in turn increases the secretion of pro-inflammatory factors such as interferon (IFN)-γ, TNF-α, IL-12, and IL-17 (18), leading to the aggravation of local intestinal inflammation and tissue damage. TL1A also promotes the expansion, survival and function of innate lymphoid cells, which play a key role in mucosal immune barrier (13, 19). Simultaneously, the TL1A/DR3 signaling activates the TGF-β/Smad pathway in fibroblasts, upregulating α-SMA expression and inducing the aberrant deposition of collagen, driving the process of intestinal fibrosis (20, 21). Studies have shown that neutralization of TL1A reduces fibroblast and myofibroblast numbers and reverses established colonic fibrosis (22). These findings position TL1A as a promising dual-effect therapeutic target for IBD, addressing inflammation and fibrosis at the same time.

TL1A is a relatively tissue-specific target, primarily expressed on endothelia cells and immune cells within intestinal lesions of IBD patients, which reduces off-target risks and enhances its therapeutic appeal (23). Several anti-TL1A monoclonal antibodies (mAbs), such as TEV-48574 (Duvakitug), PRA023 (Tulisokibart), and RVT3101 (PF-06480605), have entered clinical trials, demonstrating proof-of-concept for TL1A blockade in IBD with acceptable safety profiles (24–27). However, their clinical translation faces challenges, including short serum half-lives necessitating frequent dose regimens, limited patient eligibility, and suboptimal treatment outcomes for moderate-to-severe IBD. To address these limitations, we developed GB20-5A8-31, a novel anti-TL1A antibody engineered with an extended half-life and superior developability profile, aiming to achieve more potent, convenient, and broadly effective therapy. In this study, we developed a human IgG1 anti-TL1A antibody, GB20-5A8-31, affinity-optimized through an AI-assisted antibody engineering approach and evaluated its activities *in vitro* and *in vivo*. Our findings highlighted the unique advantages of GB20-5A8–31 over TEV-48574, such as favorable efficacy in both the 2,4,6-trinitro-benzenesulfonic acid (TNBS)-induced rat and DSS-induced hTLIA-transgenic mouse acute IBD models, and lower hydrophobicity that facilitates the development for high-concentration formulations. These attributes demonstrate its potential and provide strong support for its further clinical development.

## Materials and methods

### Antibody screening, expression, and purification

Anti-hTL1A monoclonal antibodies were generated using the hybridoma technique. Briefly, Balb/c mice (Charles River, female) were immunized intraperitoneally with recombinant human TL1A trimer protein (Kactus Biosystems, #FSF-HM415) using a primary-boosting protocol. Subsequently, B cells were collected from the spleens of the immunized mice and fused with the myeloma cells via electrofusion. Hybridoma cells were cultured in HAT media and screened using enzyme linked immunosorbent assay (ELISA) and fluorescence activated cell sorting (FACS)-based binding assays to obtain hit clones. The variable fragment sequences of the hit clones were subcloned into a human IgG1 framework for recombinant production in CHO-S cells. Finally, the antibody was purified by protein A chromatography for further studies.

### Antibody engineering: AI-assisted affinity maturation and humanization

The affinity maturation of parental molecules was conducted through the following strategy. An AI-assisted approach was implemented using a locally deployed AlphaFold3 system for antigen-antibody docking to map the precise epitope of parental molecules. First, a three-dimensional model of 5A8 was generated. This model was then docked with the crystal structure of TL1A (Protein Data Bank: 2RE9), enabling the identification of key interface residues on 5A8 that interacted with hTL1A. Targeted mutations were introduced to enhance binding energetics, then sub-cloned into a human IgG1 expression vector, expressed in CHO-S cells, and purified using protein A chromatography. As 8E3 clone already exhibited high affinity (KD = 7.64*10^-^¹^0^ M), no affinity maturation was required, and engineering efforts focused solely on humanization to reduce immunogenicity.

Humanization was performed based on parent molecules. First, the complementarity-determining regions (CDRs) of the variable area with heavy chain (VH) and light chain (VL) sequences were annotated according to the Kabat numbering scheme. Then, the Immunogenetics (IMGT) database was searched to identify human germline sequences exhibiting the highest homology to the VH and VL regions, respectively. Following this, CDR grafting was performed and critical framework residues were reverted to murine counterparts to ensure the structural stability and antigen-binding affinity.

To extend serum persistence, the candidate Abs were engineered by introducing the YTE mutation (M252Y/S254T/T256E) in the Fc region of the human IgG1 backbone. This triple amino acid substitution was introduced via site-directed mutagenesis and confirmed by Sanger sequencing (Azenta Life Sciences). The final humanized variants were expressed in CHO-S cells and purified for functional characterization.

### Surface plasmon resonance

The binding affinity between the candidate Abs and TL1A trimers (human, cynomolgus, and rat) was evaluated using a Biacore T200 surface plasmon resonance system. To mimic physiological conditions, the experiments were conducted in 1 × HBS-EP buffer (pH 7.4, 0.05% Tween-20) at 37°C. The candidate mAbs (ligands) were immobilized on a CM5 sensor chip, while recombinant hTL1A-trimer (Kactus Biosystems, #FSF-HM415), cTL1A-trimer (Kactus Biosystems, #FSF-CM115), or rTL1A-trimer (Kactus Biosystems, #FSF-RM215), serving as analytes, and were injected in a concentration gradient (100–0 nM) at a flow rate of 30 μL/minute. Real-time sensorgrams were processed using Biacore Evaluation Software v3.2. The signal is expressed in Response Units (RU), the standard quantifiable output in SPR spectroscopy, where 1 RU corresponds to a change in surface-bound mass of approximately 1 pg/mm². Binding kinetics were derived by globally fitting the data to a 1:1 Langmuir binding model. Equilibrium dissociation constants were calculated to assess cross-species reactivity.

### Flow cytometry

The hDR3-HEK293 cells were cultured in high glucose dulbecco’s modified eagle medium (DMEM, Gibco, #11965092) supplemented with 10% fetal bovine serum (FBS), 1% penicillin-streptomycin (Gibco, #15140-122). Cells were collected and washed twice with phosphate buffer saline (PBS, Hyclone, # SH30256.01). Then, 4 × 10^4^ cells/well were added to 96-well v-bottom plates. A mixture of 100 μL/well containing 25 μg/mL candidate antibodies and 5 μg/mL PE-hTL1A-trimer in FACS buffer (Thermo, #00422257) was added. The plates were incubated at 4°C for 30 minutes in the dark, followed by two washes via centrifugation at 1500 g for 3 minutes. The cells were then resuspended in 200 μL/well of FACS buffer for flow cytometry analysis (BD Biosciences). Data acquisition was performed using BD FACSDiva™ Software v8.0.1, with PE fluorescence measured in the FL2 channel (585/42 nm bandpass filter).

### Reporter gene assay

The hDR3-NFκB-Luc-Jurkat cells (stable reporter cells expressing human DR3 and NF-κB-driven luciferase) were cultured in RPMI 1640 medium (Gibco, # C11875500BT) supplemented with 10% FBS, 1% penicillin-streptomycin (Gibco, #15140-122), 3.5 μg/mL blasticidin (Gibco, #A11139-03), and 0.75 μg/mL puromycin (Gibco, #A11138-02) to maintain selection pressure. For the assay, 2 × 10^4^ cells per well were seeded in 96-well plates with 50 μL assay medium (RPMI 1640 + 1% FBS) and incubated overnight at 37 °C with 5% CO_2_. The hTL1A-trimer (Kactus Biosystems, #FSF-HM415) was diluted to 20 ng/mL in an assay medium. Candidate antibodies were serially diluted in PBS from 125 to 0.0019 nM. Then, Equal volumes (25 μL) of antibody solution and hTL1A-trimer solution were mixed in a 1:1 ratio and added into each well. After 3-hour incubation at 37°C with 5% CO_2_, 100 μL One-Lite™ Luciferase Assay Reagent (Vazyme, # DD1203-01) was added to each well. The microplate was shaken for 2 minutes on an orbital plate shaker and then left at room temperature for 13 minutes. Relative luciferase units (RLUs) were later measured using a BioTek Synergy H1 microplate reader (Biotek, #800TS) with a 1-second integration time. Dose-response curves were fitted to a four-parameter logistic model (GraphPad Prism v8.0) to determine IC_50_ values.

### Immune complex-induced TL1A activity assay in PBMCs

The PBMCs were purchased from Miltenyi Biotec (#PB050C) and adjusted to 1 × 10^6^ viable cells/mL in RPMI 1640 medium (Gibco, # C11875500BT) supplemented with 10% FBS and 1% penicillin–streptomycin (Gibco, #15140-122). Immune complex-induced TL1A activity was assessed by measuring IFN-γ expression from PBMCs. To stimulate IFN-γ production, hIL-12, hIL-18, and hTL1A were added to the PBMCs at final concentrations of 2 ng/mL, 50 ng/mL, and 100 ng/mL, respectively. The hTL1A triggered the release of IFN-γ in hIL-12/IL-18 primed PBMC cells. To evaluate the inhibition of candidate antibodies, 10 μL of serially diluted antibody solution was added to the wells. After 24-hour incubation at 37°C with 5% CO_2_, the supernatants were collected for IFN-γ assessment. And the IFN-γ levels were quantified using a human IFN-γ ELISA kit (Elabscience, #E-EL-H0108) according to the manufacturer’s protocol. Absorbance was measured at 450 nm (reference: 570 nm) on a microplate reader (BioTek Synergy H1). Dose-response curves were fitted to a four-parameter logistic model (GraphPad Prism v8.0) to determine IC_50_ values.

### Pharmacokinetic study in humanized FcRn-transgenic Sprague-Dawley rats

humanized FcRn-transgenic Sprague-Dawley (SD) rats (250–270 g, male) were purchased from Beijing Vital River Biotechnology Co., Ltd and housed in an animal facility under the supervision of IACUC (20220301002) of Shenzhen Institute for Drug Control.

The humanized FcRn-transgenic SD rats were randomized into 2 groups, which received a single subcutaneous administration of 2 mg/kg GB20-5A8–31 or TEV-48574. Blood samples were collected under anesthesia using retro-orbital bleed. And the predefined time points including 0 h (pre-dose), 2 h (Day 0), 4 h (Day 0), 24 h (Day 1), 48 h (Day 2), 72 h (Day 3), 96 h (Day 3), 120 h (Day 5), 168 h (Day 7), 240 h (Day 10), 336 h (Day 14) post-administration.

Serum was separated by centrifugation (3,000 × g, 10 minutes, 4°C) and stored at -80°C until analysis. Drug concentrations were quantified using established ELISA methods. Pharmacokinetic parameters were calculated via non-compartmental analysis (Phoenix WinNonlin v8.3).

### TNBS-induced rats acute IBD model

SD rats (250–270 g, male) were purchased from Shanghai SLAC Animal Co., Ltd and housed in an animal facility under the supervision of IACUC (KXX2401P) of Shanghai Medicilon Inc.

The SD rats (250–270 g, male) were fasted for 24 hours prior to TNBS induction. Briefly, the SD rats were anesthetized with an intraperitoneal injection of Tribromoethanol (Nanjing Aibei Biotechnology Co., Ltd, 60 mg/kg). The TNBS-ethanol solution (0.5 mL, 18 mg/mL in 40% ethanol) was administered via a suitable medical-grade catheter inserted 8 cm proximal to the anus. After dosing, rats were maintained in a head-down position for 30 minutes to ensure mucosal contact. Disease progression was monitored using the Disease Activity Index (DAI), which evaluates weight loss, stool consistency, and fecal blood (**Table 1**).

**Table 1:** Criteria for DAI scoring.

Score	Body weight loss (%)	Stool consistency	Fecal blood
0	None	Normal	None
1	≤ 5%	/	Bloody stools (Light blue)
2	6-10%	Soft adherent stool	Bloody stools (Deep blue)
3	11-15%	/	/
4	>15%	Liquid stool	Visible bloody stool

Rats were randomized to groups according to their mean DAI on Day 1. The TNBS-induced IBD rats either received no antibody treatment or intravenous injections (*i.v.*) of 10 mg/kg antibodies (GB20-5A8-31, GB20-8E3–51 or TEV-48574 (MCE, #HY-P990006) on day 1 and day 4. At the end of the study (Day 8), rats were euthanized with CO_2_ induction followed by cervical dislocation specified in the AVMA Guidelines for the Euthanasia of Animals: 2020 Edition. The colon tissue was harvested for weighting, imaging analysis by ImageJ software (NIH), and histopathology examination (H&E staining, or Masson’s trichrome staining).

### DSS-induced IBD model in hTL1A transgenic mice

Female hTL1A transgenic mice (C57BL/6 background, 6–8 weeks old) were purchased from Biocytogen (Cat# 111997), and housed in an animal facility under the supervision of IACUC (SZIDC-YL-20250820-03) of Shenzhen Institute for Drug Control.

The hTL1A transgenic mice were randomly divided into four groups (n=6): Control group, Model group, GB20-5A8–31 group, and TEV-48574 group. Except for the Control group, all other groups received 3.5% dextran sulfate sodium (DSS, YEASEN, #60316ES80) in their drinking water for 9 days to establish an acute IBD model. Starting from day 1 of DSS induction, the GB20-5A8-31 (30 mg/kg), TEV-48574 (30 mg/kg) or vehicle were administered via intraperitoneal injection every three days. Body weight changes, stool consistency, and fecal bleeding were monitored daily to calculate the disease activity index. On the experimental endpoint (day 9), mice were euthanized with CO_2_ induction followed by cervical dislocation specified in the AVMA Guidelines for the Euthanasia of Animals: 2020 Edition. The colon tissue was collected for length measurement and photography. A portion of the colon tissue was taken for histopathological scoring (H&E staining and Masson’s trichrome staining).

### Histopathology analysis

Colon tissues were fixed overnight in 10% neutral formalin, embedded in paraffin, and cut into 5 μm sections of tissue samples. Following deparaffinization, sections were stained with hematoxylin and eosin (H&E) or Masson’s trichrome using standard methods. Representative photomicrographs were captured under 4× magnification using a microscope. The histopathological evaluation of colonic ulceration areas was conducted by two pathologists following scoring scale (**Tables 2**, **3**).

**Table 2:** Pathological score of H&E staining.

Parameter	0	1	2	3	4
Epithelial integrity	Intact	Focal erosion/loss	Extensive erosion	Focal ulceration	Extensive ulceration
Inflammatory infiltrate	None	Mild (<25% of field)	Moderate (25–50%)	Severe (>50%)	Transmural infiltration
Mucosal edema	None	Mild	Marked	With submucosal widening	Separation of mucosa and muscle
Crypt damage	None	Partial dilatation	<50% crypt loss	≥50% crypt loss	Crypt loss
Goblet cell depletion	None	Mild reduction	Moderate reduction	Marked reduction	Almost complete loss

**Table 3:** Pathological Score of Masson’s trichrome staining.

Parameter	Collagen deposition area
0	None
1	<5%
2	5–10%
3	10–20%
4	20–30%
5	>30%

### Developability assessment of GB20-5A8-31

The biophysical properties of GB20-5A8–31 and its stability under stress conditions including freeze-thaw cycles (-80°C for 24 h and 25°C for 24 h, three times), elevated temperatures (40°C for seven days), light exposure (4500lux ± 500lux, seven days), oxidation (1mM H_2_O_2_, seven days), and pH variations (pH5.5 or 8.5, seven days) were characterized through the following analytical approaches. Abs solubility index was assessed via hydrophobic interaction chromatography (HIC) employing a Protemix HIC Butyl-NP5 column. Thermal stability was evaluated using differential scanning fluorimetry (DSF) with a Nano DSF system (Prometheus NT48). Molecular purity was determined by size exclusion chromatography (SEC) on BioCore SEC-300 (NanoChrom, China). Non-reducing capillary electrophoresis with sodium dodecyl sulfate (nrCE-SDS) was conducted on a Proteome Lab PA 800 Plus system (AB Sciex, Redwood City, CA). Binding affinity toward human TL1A-trimer (hTL1A-His) was quantified through bio-layer interferometry measurements using a Sartorius Octet^®^ R8 platform. Details of experimented processes were included in Supplementary Materials.

### Statistical analysis

Data are presented as the mean ± standard error of the mean (SEM). The sample size (n) for each *in vivo* experiment is indicated in the figure legends. For longitudinal data (e.g., time-course measurements), statistical significance was assessed by two-way repeated measures ANOVA followed by Dunnett’s *post hoc* test at individual time points. For comparisons among multiple groups at a single endpoint, one-way ANOVA followed by Dunnett’s multiple comparisons test was used. All analyses were performed using GraphPad Prism software (version 8.0.1; GraphPad Software, Inc., San Diego, CA). The significance levels are denoted as ∗ *p* < 0.05, ∗∗ *p* < 0.01, ∗∗∗ *p* < 0.001.

## Results

### Generation of functionally active monoclonal antibodies to human TL1A by hybridoma technology

**Figure 1:**
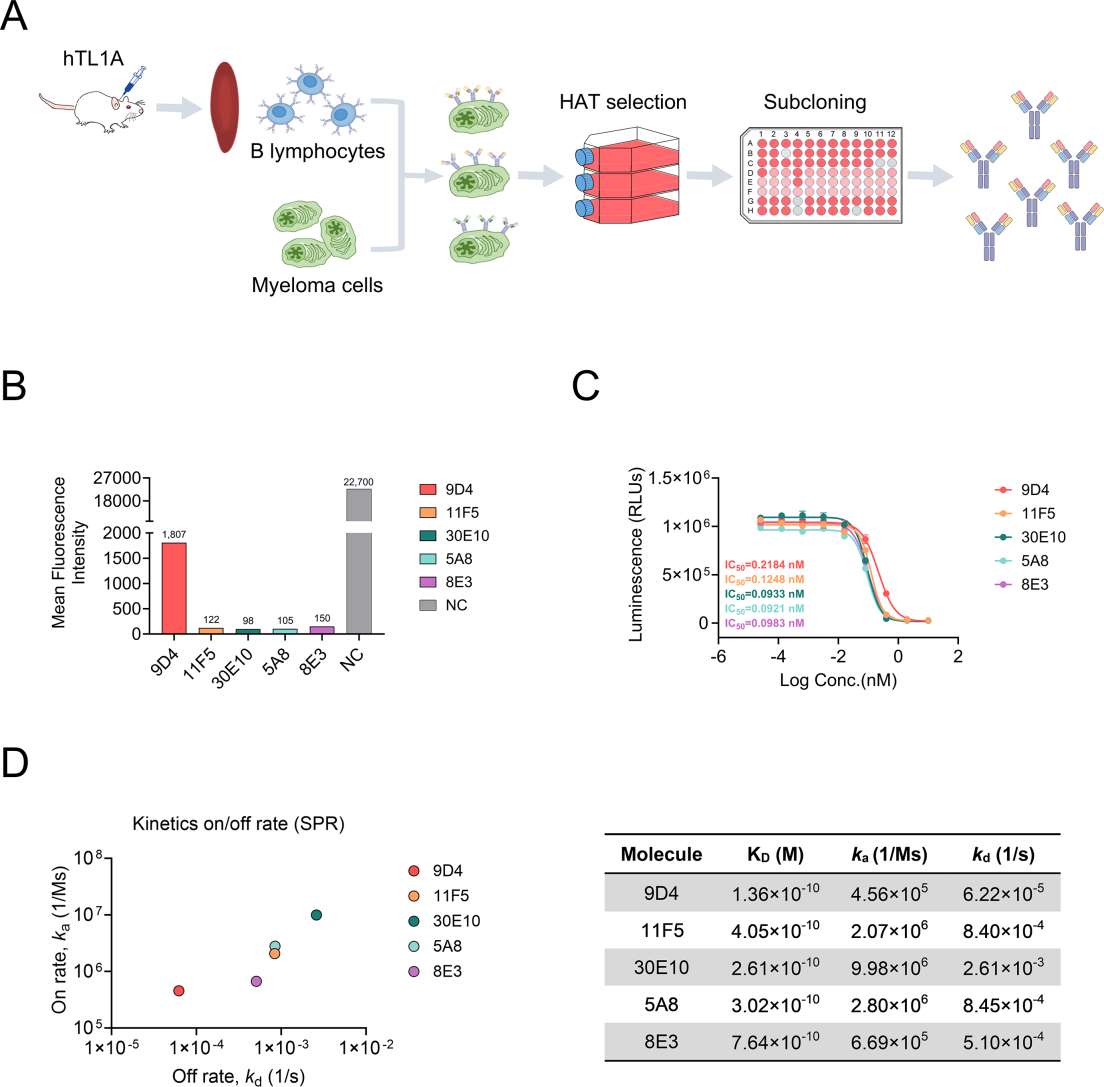
Generation and characterization of anti-TL1A antibodies. **(A)** Schematic illustration of hybridoma-based production of recombinant anti-TL1A antibodies. **(B)** Blocking activity of candidate antibodies against hTL1A-DR3 interaction by FACS, NC: negative control. **(C)** Reporter gene assay demonstrating inhibition of the TL1A/DR3 reporter activity by candidate antibodies. **(D)** Binding affinity of anti-TL1A antibodies to hTL1A as determined by SPR. K_D_ values were determined by global fit using 1:1 binding model. Dose-response curves were fitted with a four-parameter logistic model. IC_50_ values are reported as the concentration producing 50% of the maximum response together with 95% confidence intervals.

Antibodies against human TL1A (hTL1A) were generated in-house using mouse hybridoma technology (**Figure 1A**). An initial screen yielded 68 monoclonal hybridomas expressing anti-TL1A antibodies. The unique sequences encoding the Fab region were fused to human IgG1 Fc for recombinant expression and *in vitro* activities. Neutralizing activities against hTL1A were assessed using fluorescence-activated cell sorting (FACS) (**Figure 1B**). At the same concentration, clones 11F5, 30E10, 5A8, and 8E3 showed similar neutralizing abilities, whereas clone 9D4 was less active. Cell-based functional activities were evaluated using an NF-κB-driven luciferase reporter assay in Jurkat cells overexpressing human DR3. Results indicated that all tested antibodies effectively inhibited NF-κB activation with similar IC_50_ values, and clones 30E10, 5A8, and 8E3 demonstrated similar efficacy (**Figure 1C**). The affinity of the antibodies for hTL1A was tested by surface plasmon resonance (SPR). As shown in **Figure 1D**, all antibodies exhibited a high affinity for hTL1A with equilibrium dissociation constant (K_D_) ranging from 1.36×10–^10^ to 7.64×10–^10^ M. Among them, clone 30E10 dissociated more rapidly (2.61×10–^3^ s^-1^). Based on *in vitro* activities and affinities, clones 5A8 and 8E3 were selected for further development.

### AI-assisted affinity maturation and humanization

**Figure 2:**
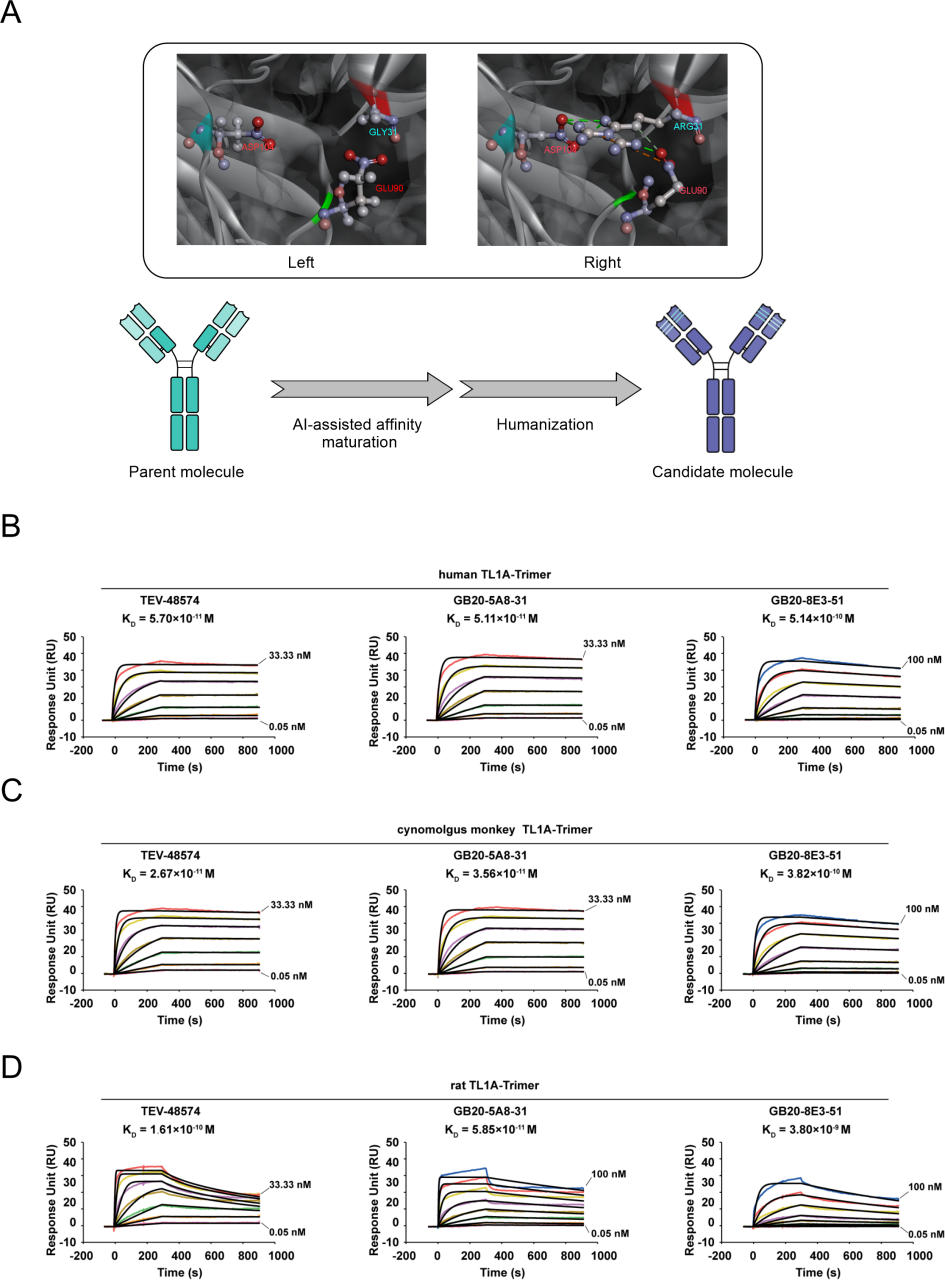
Humanization and AI-assisted affinity maturation. **(A)** AI-assisted structure modeling and schematic diagram of the optimization of anti-TL1A antibodies. An AI tool, AlphaFold3, was used to predict the 5A8-TL1A complex. The distance between Gly31 (HCDR1) and Glu90 (TL1A) was measured at 3.8 Å (left), when Gly31Arg mutation was introduced, Arg31 formed robust inter-molecular interactions with Glu90 and Asp104 (right). **(B–D)** Binding affinities of GB20-5A8–31 and GB20-8E3–51 to human TL1A. GB20-5A8–31 and GB20-8E3–51 were generated from clones 5A8 and 8E3, respectively. **(B)**, cynomolgus monkey TL1A **(C)**, and rat TL1A **(D)** were determined by SPR. RU (response unit): The standard signal unit for SPR detection that reflects changes in the mass of molecules bound to the chip surface. Sensorgrams (colored lines) are overlaid with global fits to a 1:1 interaction model (black lines).

To improve affinity and reduce immunogenicity, the antibodies underwent affinity maturation through AI-assisted structure modeling and mutation, followed by humanization (**Figure 2A**). Molecular docking simulation between 5A8 and hTL1A was performed, and structural analysis of the docked pose showed that residue Gly31 on HCDR1 of 5A8 is in close proximity to residues Glu90/Asp104 of hTL1A, though no significant interactions were observed. To enhance affinity to human TL1A, a Gly31Arg mutation was introduced to 5A8. Structure modeling showed that the side chain of Arg31 formed a network of robust intermolecular interactions with Glu90 and Asp104 on human TL1A, including salt bridges and hydrogen bonds, with calculated interaction energies of -15.26 kcal/mol and -24.77 kcal/mol for each residue, respectively. After humanization, the antibody GB20-5A8–31 was obtained, whose binding kinetics were then evaluated using SPR. GB20-5A8–31 had a K_D_ value of 5.11×10–^11^ M, representing a six-fold improvement in affinity compared to the parental clone (**Figure 1D** and 2B). GB20-8E3-51, the humanized version of clone 8E3 (**Figure 2A**), showed a slight decrease in affinity compared to its parent, with a K_D_ value of 5.14×10–^10^ M (**Figures 1D**, **2B**). The hTL1A-binding affinity of both antibodies was comparable to TEV-48574. Furthermore, both GB20-5A8–31 and GB20-8E3–51 displayed excellent cross-reactivity for TL1A from cynomolgus monkeys and rats (**Figures 2C, D**), making them desirable candidates for nonclinical studies in various animal models.

### GB20-5A8–31 and GB20-8E3–51 effectively blocked NF-κB signaling and reduced IFN-γ production *in vitro*

**Figure 3:**
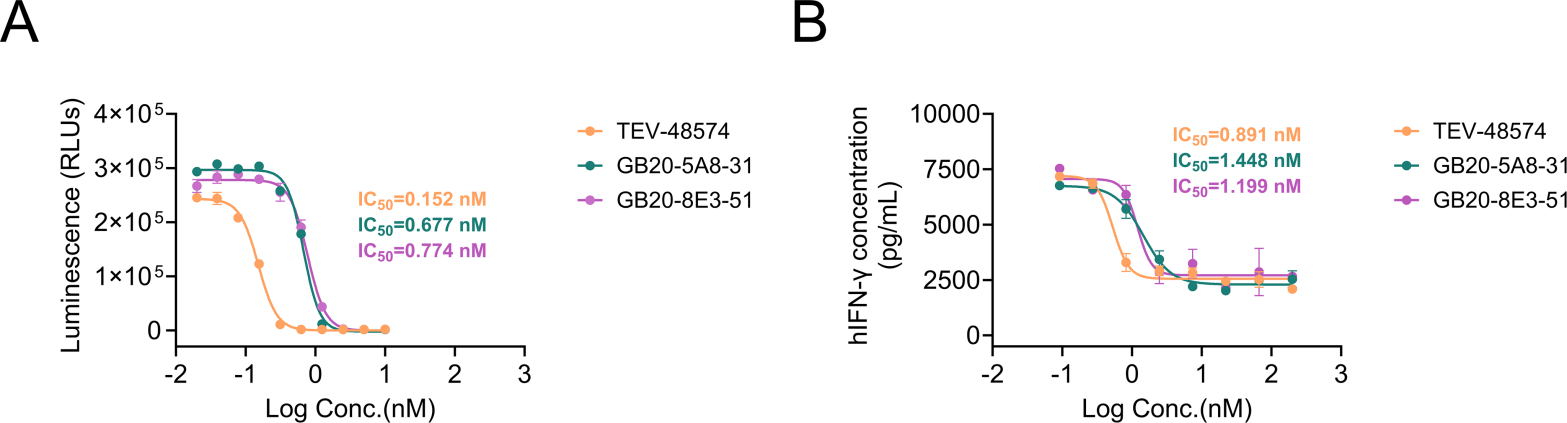
GB20-5A8–31 and GB20-8E3–51 effectively blocked the TL1A/DR3 signaling pathways *in vitro*. **(A)** Blocking activity in hDR3-NFκB-Luc-Jurkat cells. Jurkat reporter cells stably expressing human DR3 and NF-κB-driven luciferase were seeded in 96-well microtiter plates at a density of 20,000 cells per well. The cells were then activated with a final concentration of 20 ng/mL of hTL1A trimer, then incubated with serially diluted candidate molecules for three hours. Luciferase activity (RLUs) was quantified and demonstrated dose-dependent inhibition. **(B)** Inhabitation of TL1A-induced production of IFN-γ by anti-TL1A antibodies in PBMCs. IL-12/IL-18-activated PBMCs were treated with antibodies for 24 hours. The concentration of IFN-γ was subsequently measured by ELISA. Dose-response curves were fitted with a four-parameter logistic model. IC_50_ values are reported as the concentration producing 50% of the maximum response together with 95% confidence intervals.

Since TL1A binds to its receptor DR3 and activates downstream signaling pathways to exert pro-inflammatory effects, we assessed the *in vitro* neutralizing activity of the candidate antibodies using reporter gene assays in hDR3-NFκB-Luc-Jurkat cells. Both GB20-5A8–31 and GB20-8E3–51 dose-dependently inhibited the expression of luciferase driven by an NF-κB response element, with IC_50_ values of 0.677 nM and 0.774 nM, respectively, indicating effective blockade of the TL1A/DR3 signaling pathway (**Figure 3A**). The ability of GB20-5A8–31 and GB20-8E3–51 to inhibit TL1A function were further tested in peripheral blood mononuclear cells (PBMCs), where TL1A binding to DR3 synergistically enhanced IFN-γ production in IL-12/IL-18 primed PBMCs. Both antibodies suppressed IFN-γ expression with IC_50_ values of 1.448 nM for GB20-5A8–31 and 1.199 nM for GB20-8E3-51 (**Figure 3B**), effectively blocking TL1A/DR3-mediated Th1 responses. Collectively, these *in vitro* cell-based assays confirmed that both antibodies exerted their anti-inflammatory effects by targeting the TL1A/DR3 axis and modulating Th1 immune responses.

### GB20-5A8–31 and GB20-8E3–51 potently ameliorates disease pathology in TNBS-induced acute IBD model in rats

**Figure 4:**
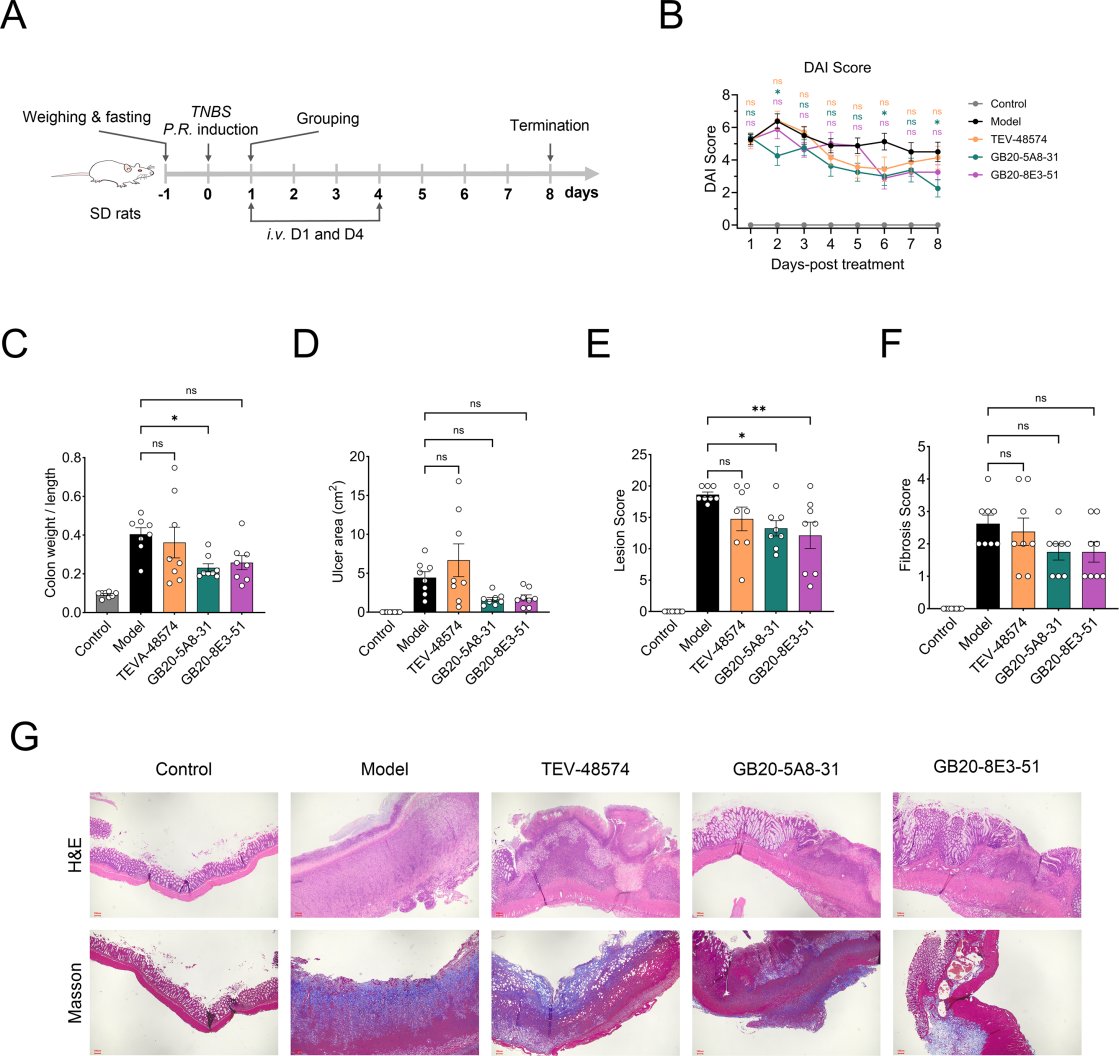
GB20-5A8–31 significantly ameliorate TNBS-induced acute IBD in SD rats. SD rats were fasted for 24 hours, and TNBS-ethanol solution (18 mg/mL) was administered rectally. The next day, the rats were grouped and administered with candidate antibody molecules (*iv.*, 10 mg/kg). **(A)** The schedule of TNBS-induced acute IBD model and drug treatment in SD rats (n = 8). **(B)** The disease activity index (DAI) score was examined on days 1 to 8. **(C)** The colon weight/length and ulcer area of colon **(D)** were counted. **(E, F)** Pathological score of colon specimens. **(G)** Representative H&E staining (top) and Masson’s trichrome staining (bottom) of colon specimens. Original magnification, 10×of H&E staining and 4× of Masson’s trichrome staining. Scale bar: 100 μm. All data are expressed as the mean ± SEM. *p < 0.05, **p < 0.01, ns, no significance.

To evaluate the effect of TL1A neutralization on mucosal inflammation, we established a TNBS-induced acute rat IBD model (28, 29). The experimental protocol involved intravenous administration of GB20-5A8-31, GB20-8E3-51, or TEV-48574 (10 mg/kg) on day 1 and day 4 (**Figure 4A**). Both GB20-5A8-31- and GB20-8E3-51-treated group demonstrated superior therapeutic efficacy across multiple parameters. And GB20-5A8–31 significantly reduced Disease Activity Index (DAI) scores compared to the model group (*p* < 0.05, **Figure 4B**), indicating improvement in clinical signs in TNBS-induced rats. Moreover, GB20-5A8-31-treated groups significantly lowered the colon weight/length ratios (*p* < 0.05, **Figure 4C**), and also showed a statistically significant difference in the histopathological score (p < 0.05, **Figures 4E, G**), suggesting its role in alleviating inflammatory response and tissue damage. In the GB20-8E3–51 treated group, the pathological score decreased compared with the model group, with a statistically significant difference (P < 0.01, **Figures 4E, G**), with reduced inflammatory cell infiltration, necrosis, edema within or beneath the colonic mucosa, while no significant improvement was observed in the remaining indicators, indicating a certain ameliorative effect of this drug on colonic tissue inflammatory injury. As a competitor control, TEV-48574 showed no statistically significant differences compared with the model group in all observed indicators (**Figures 4B–G**). In addition, no statistically significant differences were observed in ulcer area or fibrosis scores across any groups (**Figures 4D, F, G**). These findings suggested that GB20-5A8–31 was clearly effective in treating TNBS-induced acute IBD, significantly improving disease activity, gross colonic morphology and histopathological inflammatory damage. GB20-8E3–51 exhibited only modest anti-inflammatory properties, whereas TEV-48574 displayed no notable therapeutic efficacy in this model.

### GB20-5A8–31 ameliorates DSS-induced acute IBD in hTL1A transgenic mice via suppression of inflammation

**Figure 5:**
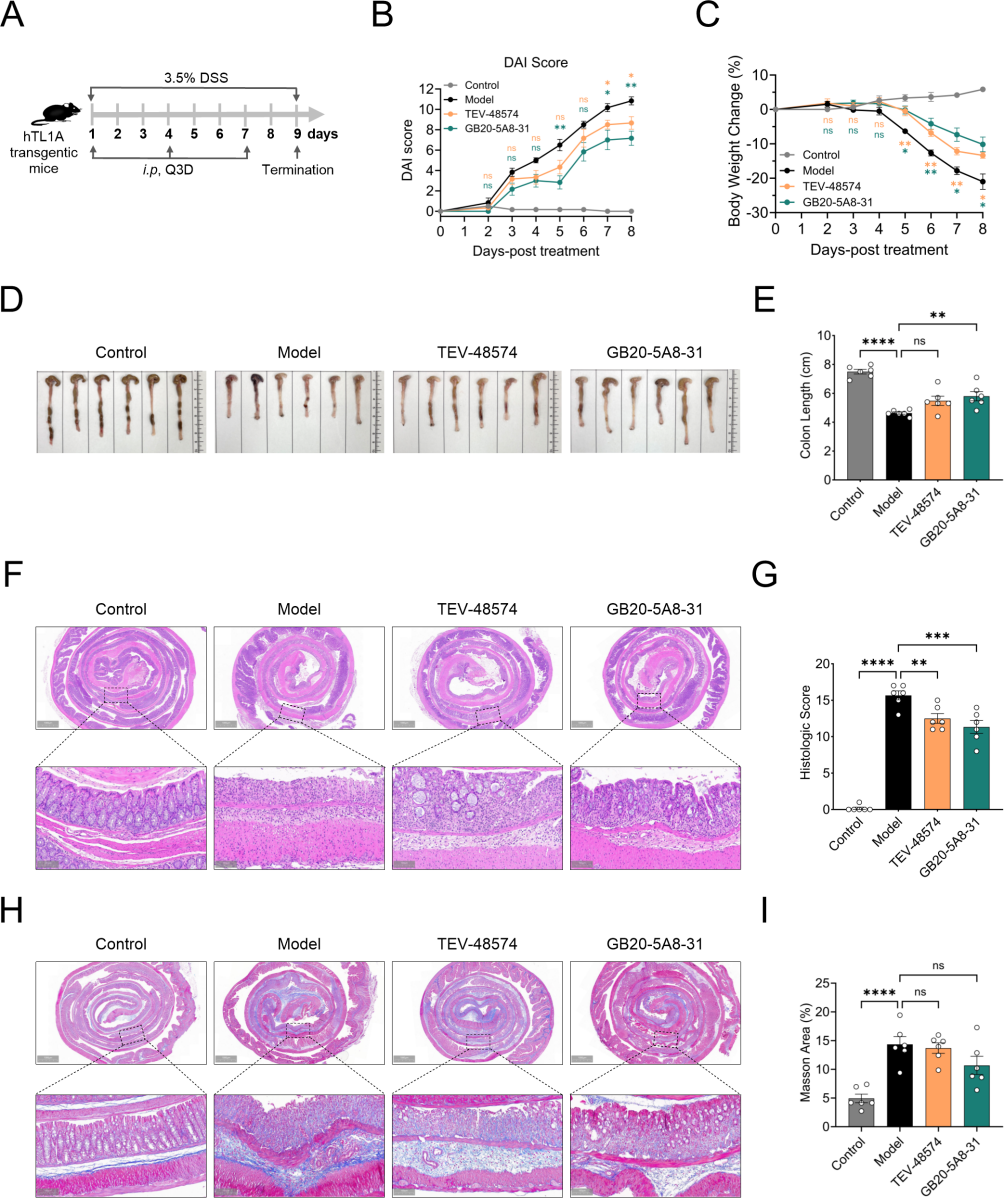
GB20-5A8–31 alleviates DSS-induced acute IBD in hTL1A transgenic mice. The hTL1A transgenic mice were received 3.5% dextran sulfate sodium (DSS) in their drinking water for 9 days to establish an acute IBD model. **(A)** The schedule of DSS-induced acute IBD model and drug treatment (n = 6). **(B)** The disease activity index (DAI) score was examined on days 1 to 9. **(C)** The body weight change was examined over 9 days. **(D, E)** Representative colon photographs and colon length measurements. **(F, G)** Representative H&E staining and Histopathological scores of colon specimens. **(H, I)** Representative Masson’s trichrome staining of colon specimens. Original magnification, 2×and 10×. Scale bar: 1000 μm and 200 μm. All data are expressed as the mean ± SEM. *p < 0.05, **p < 0.01, ***p < 0.001, ****p < 0.0001, ns, no significance.

To further verify the efficacy of GB20-5A8-31, we investigated it in a DSS-induced acute IBD model in human TL1A transgenic mice (**Figure 5A**). Our findings indicate that GB20-5A8–31 exhibited favorable therapeutic efficacy in treating acute IBD compared to TEV-48574. Specifically, GB20-5A8–31 outperformed TEV-48574 in improving the DAI score, although both compounds promoted weight recovery (**Figures 5B, C**). In addition, compared to the model group, the GB20-5A8–31 treatment group showed a more pronounced improvement in colon shortening (*p* < 0.01), while the TEV-48574 treatment group showed no statistically significant differences (**Figures 5D, E**). Histological evaluation revealed that both GB20-5A8–31 and TEV-48574 significantly reduced inflammatory cell infiltration and epithelial damage in intestinal tissues, with GB20-5A8 31 treatment demonstrating more pronounced improvement (**Figures 5F, G**). Regarding anti-fibrotic activity, the fibrotic area in the TEV-48574-treated group was comparable to that in the model group. The fibrotic area in the GB20-5A8-31-treated group was reduced, showing a slight improvement trend, but no statistically significant difference was observed between the two groups compared to the model group (**Figures 5H, I**), Collectively, these findings suggest that GB20-5A8–31 offers beneficial effects in ameliorating disease activity, limiting intestinal structural damage, and modulating inflammatory responses.

### Pharmacokinetic studies of GB20-5A8–31 in humanized FcRn-transgenic rats

**Figure 6:**
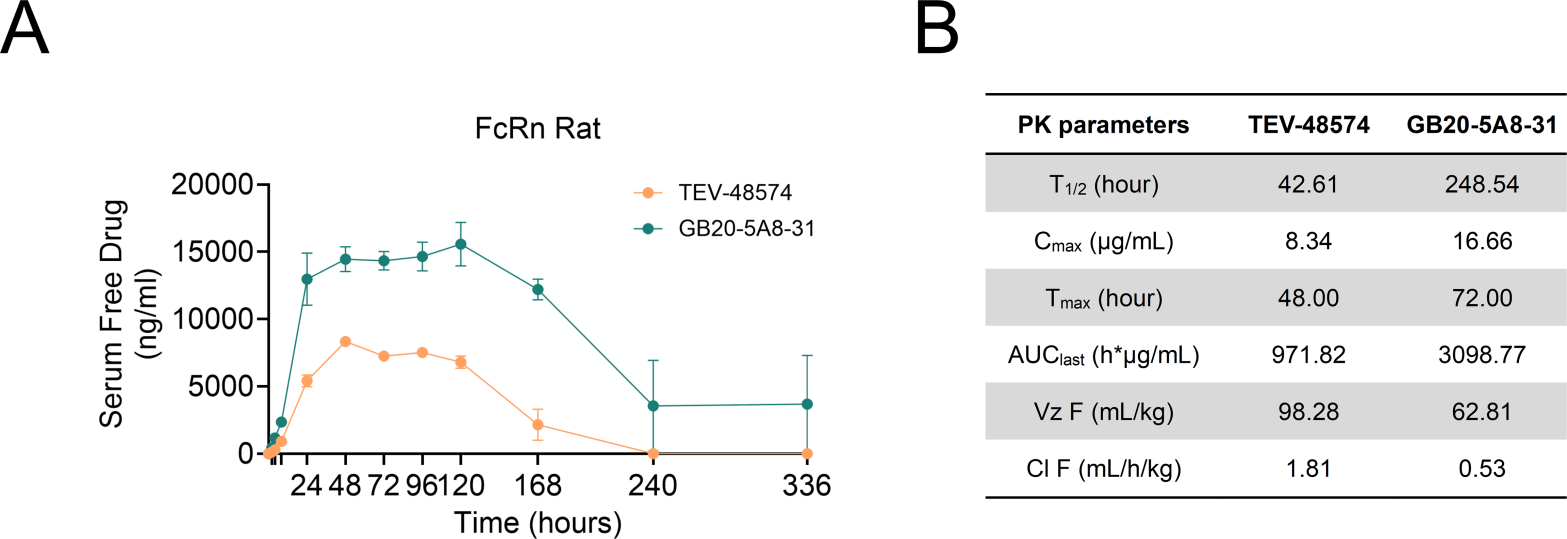
Pharmacokinetic analysis of GB20-5A8–31 in humanized FcRn-transgenic SD rats. TEV-48574 or GB20-5A8–31 was injected subcutaneously into humanized FcRn-transgenic SD rats (n = 3, 2 mg/kg). **(A)** Serum was collected at different time points to measure the drug concentration by ELISA. **(B)** The pharmacokinetic parameters were subsequently calculated. Data are expressed as mean ± standard error of the mean (SEM).

To extend serum persistence, the GB20-5A8–31 was engineered by introducing the YTE mutation (M252Y/S254T/T256E) in the Fc region. These substitutions increase affinity for human FcRn at acidic pH while preserving minimal binding at neutral pH, a mechanism known to prolong IgG recycling and systemic half-life (30, 31). The pharmacokinetic properties of GB20-5A8–31 were tested in humanized FcRn transgenic rats. Following single-dose subcutaneous administration (2 mg/kg), GB20-5A8–31 had a half-life (T_1/2_) of 248.54 hours, a peak concentration (C_max_) of 16.66 µg/mL, total systemic exposure (AUC_last_) of 3098.77 µg·h/mL and a clearance rate (CL/F) of 0.53 mL/h/kg. In contrast, TEV-48574 had a T_1/2_ of 42.61 hours, a C_max_ of 8.34 µg/mL, and a CL/F of 1.81 mL/h/kg, indicating faster clearance (**Figures 6A, B**). These findings suggest that GB20-5A8–31 exhibits superior pharmacokinetic properties in the context of human-relevant FcRn, particularly an extended half-life, which may allow for reduced dosing frequency in subsequent efficacy studies.

### Comprehensive developability profiling of GB20-5A8-31

**Figure 7:**
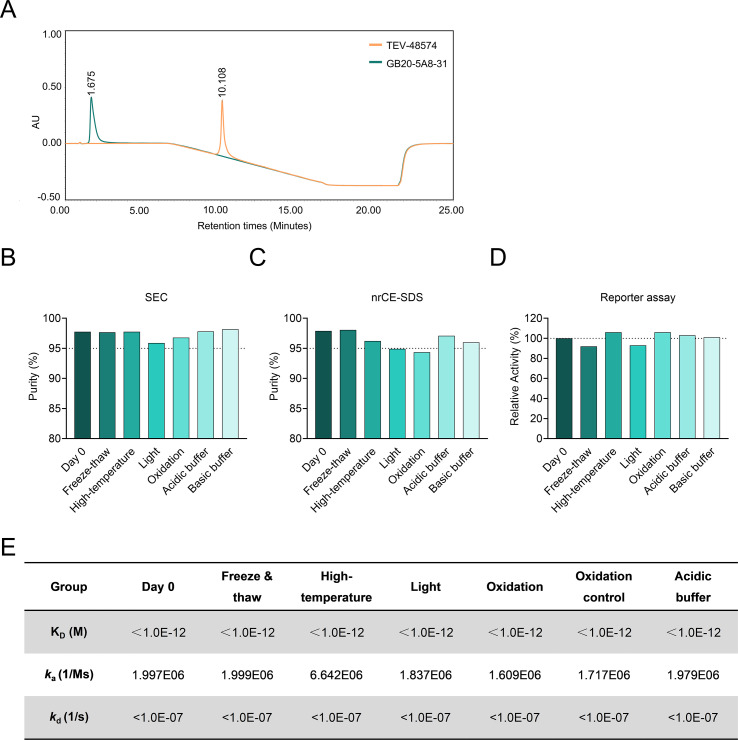
Developability assessment of GB20-5A8-31. **(A)** Hydrophobicity analysis by hydrophobic interaction chromatography (HIC). **(B-F)** Stability evaluation of GB20-5A8–31 under stress conditions. Two time points (0 and 7 days) were chosen to investigate the effects of freeze-thaw cycles, high temperature, light exposure, oxidation, and pH changes on molecular properties. The collected samples were examined for purity by SEC **(B)** and nrCE-SDS **(C)**, relative *in vitro* activity **(D)** and affinity by BLI **(E)**.

As a critical de-risking strategy for clinical translation, we conducted a systematic developability assessment of GB20-5A8-31. First, surface hydrophobicity of an antibody is a major determinant of solubility, stability and aggregation, and can be analyzed by hydrophobic interaction chromatography (HIC). As shown in **Figure 7A**, GB20-5A8–31 eluted much earlier than TEV-48574 (1.675 minutes *vs.* 10.108 minutes), indicating a less exposed hydrophobic surface of GB20-5A8–31 and a reduced tendency to form aggregates. This makes GB20-5A8–31 suitable for high-concentration formulation development—facilitating subcutaneous clinical administration and improving patient compliance. Next, differential scanning fluorimetry (DSF) revealed favorable thermal stability with a single melting transition of GB20-5A8-31(T_onset_ = 54.6°C, T_m1_ = 65.4°C, **Supplementary Table 3**). This characteristic indicates that the antibody can tolerate the thermal stress during large-scale manufacturing, reducing the risk of conformational inactivation and aggregation-induced yield loss.

For comprehensive developability assessment, GB20-5A8–31 was subjected to a series of accelerated and stress tests, including freeze-thaw cycles, elevated temperature, light exposure, oxidative stress, acidic and basic pH conditions, followed by analysis of the purity, target-binding, and biological activities post-stress. Overall, GB20-5A8–31 exhibited exceptional stability across multiple conditions. The purity of the antibody remained high with monomericity > 95% across all test conditions by size exclusion chromatography (SEC). The same trend was seen in non-reducing capillary electrophoresis with sodium dodecyl sulfate (nrCE-SDS), except for a slight reduction below 95% in purity for samples under oxidation stress (94%) (**Figures 7B, C**). It should be noted that light exposure resulted in a larger decrease in the %main peak of GB20-5A8–31 as revealed by both SEC and nrCE-SDS, suggesting that the antibody should be protected from light during storage. The activity of GB20-5A8–31 was evaluated by luciferase reporter gene assay for functional readout and by BLI for target binding affinity. Both the relative activity and the binding kinetics of the antibody were essentially unaffected after the stress treatments, indicating excellent stability (**Figures 7D, E**). The mass spectrometry results demonstrated that the intact and reduced molecular weights of GB20-5A8–31 are consistent with the theoretical values with acceptable mass accuracy (mass error is within 50 ppm, **Supplementary Tables 1**, **2**).

This integrated dataset establishes GB20-5A8–31 as a development-ready therapeutic antibody with exceptional conformational stability, low aggregation propensity, and stress-resistant biological functionality – key attributes supporting its progression into preclinical development.

## Discussion

IBD is a global health challenge with an increasing incidence, particularly in newly industrialized countries (32). Recent epidemiologic studies estimate that more than 6.8 million people worldwide suffer from IBD, generating an enormous socioeconomic burden. Despite advancements in therapeutic strategies, IBD remains a significant clinical challenge.

Developing neutralizing monoclonal antibodies (mAbs) against TL1A represents a promising strategy in IBD treatment. Several anti-TL1A mAbs are currently in clinical development, demonstrating encouraging efficacy and safety. For instance, Tulisokibart, a humanized IgG1-κ monoclonal antibody targeting TL1A, has demonstrated significant efficacy and a favorable safety profile in both UC and CD patients in clinical trials (26). Treatment with RVT-3101, a fully human IgG1 monoclonal antibody targeting TL1A, resulted in improved clinical remission and endoscopic improvement in patients with UC in a phase 2 trial (24, 33). Duvakitug is a human IgG1-λ2 monoclonal antibody targeting TL1A developed by TEVA. In a recent publication of data from a Phase 2b study of Duvakitug in patients with UC and CD, promising efficacy and safety in different dose groups (34) were presented. In adults diagnosed with moderate-to-severe UC, the high-dose arm (900 mg) achieved a placebo-adjusted clinical remission rate of 27.4% (p=0.003), outperforming RVT-3101 (20%) and Tulisokibart (25%), suggesting the potential for best-in-class efficacy. In the CD cohort, a 35% placebo-adjusted clinical remission rate (p<0.001) was observed in the high-dose group, achieving the primary endpoint. Furthermore, among patients with prior conventional therapy failure, clinical remission rates of Duvakitug-treated group reached 44% in UC and 29% in CD. These findings suggest that anti-TL1A antibodies may improve the prognosis of patients who had failed prior conventional treatment. All dose groups exhibited adequate tolerability with no emergent safety signals. While phase 3 readout on the efficacy and safety for patients with active UC and CD is eagerly anticipated to support the role of TL1A/DR3 signaling in chronic inflammation, the available clinical data convinced us to develop our TL1A-targeted biologics.

Traditional antibody engineering relies on low-throughput screening, which is inefficient and can cause mutations that compromise protein stability. To address these limitations, we adopted a structure-based AI-assisted rational design strategy. Leveraging the 3D structure of the antibody-antigen complex and quantitative binding free energy (ΔG) calculations, integrated with molecular dynamics simulations, this approach rationally identified optimal mutations that boost affinity while preserving stability. The optimized antibody GB20 showed a 6-fold higher antigen-binding affinity than the parental antibody in SPR assays and demonstrated excellent physicochemical properties in developability assessments. This AI-assisted method enables a paradigm shift in antibody engineering from “large-scale random screening” to “small-scale precision design, fundamentally improving optimization efficiency and outcome controllability.

Favorable pharmacokinetic properties are important for clinical applicability. To extend serum half-life, we introduced the YTE modification (M252Y/S254T/T256E) into the Fc domain of GB20-5A8-31, which enhances pH-dependent binding to the neonatal Fc receptor (FcRn) and promotes antibody recycling (30, 31). To accurately assess the PK impact of this engineering in a human-relevant context, we used humanized FcRn-transgenic SD rats, since they closely approximate human FcRn biology and provides data with higher translational value than wild-type rodents. Non-compartmental analysis gave GB20-5A8–31 a terminal half-life of 248.5 h (Cl F: 0.53 mL/h/kg) versus 42.61 h (Cl F: 1.81 mL/h/kg) for TEV-48574. This marked difference in half-life directly informs clinical dosing strategy by providing a strong preclinical rationale for exploring less frequent administration intervals for GB20-5A8–31 in future clinical trials, aiming to improve patient adherence and treatment outcomes.

Mechanistically, TL1A activates NF-κB signaling pathways through binding to DR3, and induces pro-inflammatory cytokine production. TL1A amplifies the T cell responses by synergizing with pro-inflammatory signals, such as amplifying Th1 responses by synergizing with IL-12 and IL-18 to upregulate IFN-γ production from Th1 cells. Functional validation demonstrated that GB20-5A8–31 potently inhibits TL1A/DR3 signaling in NF-κB-driven reporter assays (IC_50_ = 0.677 nM) and suppresses TL1A-triggered production of IFN-γ (IC_50_ = 1.448 nM), highlighting its efficacy in inhibiting the TL1A-triggered T cell response amplification. Our *in vitro* functional studies validated the role of GB20-5A8–31 in disrupting both proximal signaling events and distal inflammatory responses.

The therapeutic efficacy of GB20-5A8–31 was systematically evaluated in two well-established acute IBD models: TNBS-induced acute IBD in rats and DSS-induced acute IBD in hTL1A transgenic mice. Consistent findings were observed across both models. Compared to TEV-48574, currently the most effective clinical treatment, GB20-5A8–31 significantly improved DAI scores, gross colonic morphology, and alleviated inflammatory cell infiltration in the intestinal mucosa as assessed by histopathological analysis. These results collectively confirm the anti-inflammatory activity of GB20-5A8–31 in acute intestinal inflammation, which aligns with the therapeutic goals of targeting TL1A-DR3 signaling in IBD. The enhanced performance of GB20-5A8–31 observed in rats with TNBS-induced acute IBD is likely attributable to two synergistic factors (1): Affinity differences: GB20-5A8–31 had a higher affinity for rat TL1A (K_D_ = 5.85 ×10–^11^ M *vs.* 1.61 ×10–^10^ M), and a slower dissociation rate (1.37 ×10–^4^ s^-1^*vs.* 1.10 ×10–^3^ s^-1^), potentially prolonging the target inhibition and enhancing efficacy (2). Differences in PK properties: GB20-5A8–31 demonstrated superior PK characteristics in hFcRn-transgenic SD rats, with a lower clearance rate and longer half-life (Cl F: 0.53 mL/h/kg, T_1/2_: 248.5 h), indicating an extended duration in the body to exert its effects.

In assessing potential anti-fibrotic activity, GB20-5A8–31 treatment exhibited a non-significant trend toward reduced fibrotic area in the colonic tissue of both acute models. This preliminary observation should be interpreted with caution, as the short duration and primary inflammatory focus of acute IBD models limit their ability to evaluate anti-fibrotic efficacy. The TNBS- and DSS- induced IBD model are well-established for studying acute intestinal inflammation. The TNBS model elicits a robust Th1-mediated response, characterized by rapid, acute mucosal inflammation but minimal spontaneous fibrosis within the short experimental timeframe (28, 35, 36). Similarly, even in our hTL1A transgenic setting, the DSS-induced IBD models primarily recapitulate acute epithelial damage and inflammatory infiltration. A key limitation of both models is their inability to faithfully model the chronic, relapsing inflammation and the subsequent progressive fibrosis that are hallmarks of human IBD. Fibrosis is a sequel of sustained tissue injury and requires a prolonged timeframe for the activation of pro-fibrotic pathways and extracellular matrix deposition (37, 38). Therefore, while these acute models are highly reproducible and sensitive for evaluating anti-inflammatory efficacy, they are suboptimal for definitively assessing anti-fibrotic activity. A rigorous evaluation of GB20-5A8-31’s potential to inhibit or reverse intestinal fibrosis necessitates future studies in chronic, fibrosis-prone models, such as TNBS-induced chronic colitis, repeated low-dose DSS-induced chronic colitis, or IL-10 knockout mice, which more accurately replicate the persistent inflammatory response and progressive fibrotic remodeling observed in patients. These models will be critical for definitively assessing whether GB20-5A8–31 can inhibit key pro-fibrotic processes, including myofibroblast activation and excessive extracellular matrix deposition, and potentially reverse established fibrosis. Furthermore, exploring combination therapies with existing anti-fibrotic agents represents a promising avenue for achieving synergistic efficacy.

In addition to effectiveness, the developability of GB20-5A8–31 is a key focus in our research. Our study shows GB20-5A8–31 exhibits low hydrophobicity, significantly better than TEV-48574 (**Figure 6A**). This reduces the immunogenicity risk of GB20-5A8-31, meets stringent quality standards for clinical drugs, and enhances the potential for developing highly concentrated formulations. Meanwhile, GB20-5A8–31 is also stable under various stress challenges, maintaining structural and functional integrity. These critical attributes provide fundamental support for the GB20-5A8-31’s advancement into preclinical development and translate directly into clinical advantages. For instance, high conformational stability prolongs its *in vivo* half-life, potentially reducing dosing frequency and improving patient compliance in clinical settings.

When systematically compared with the clinical-stage anti-TL1A benchmark TEV-48574, GB20-5A8–31 demonstrates a differentiated profile with potential advantages across key dimensions. In terms of efficacy, GB20-5A8–31 induced a more pronounced suppression of disease activity and inflammation in our preclinical models. Pharmacokinetically, it exhibits an approximately 5.8-fold longer elimination half-life (248.5 hours vs. 42.61 hours), which may imply a potential reduction in dosing frequency during clinical trials. Regarding developability, its superior properties—including high solubility and low aggregation propensity—facilitate the development of a patient-friendly, high-concentration subcutaneous formulation. This balanced improvement across efficacy, pharmacokinetics, and developability positions GB20-5A8–31 as a highly promising treatment option for patients with IBD.

## Data Availability

The original contributions presented in the study are included in the article/**Supplementary Material**. Further inquiries can be directed to the corresponding author.

## Supplementary material

Supplementary Figure 1 The images of the colon at the endpoint of TNBS-induced rat IBD model.

Supplementary Figure 2 Epitope binning analysis using Bio-Layer Interferometry (BLI). HIS1K biosensor tips were first loaded with 0.3 nM human TL1A, followed by sequential association with mAb1 and mAb2. **(A)** GB20-5A8–31 was applied as mAb1, subsequent association with either GB20-5A8–31 or TEV-48574 as mAb2. **(B)** TEV-48574 served as mAb1, the subsequent introduction of either GB20-5A8–31 or TEV-48574 as mAb2.
