# Supplementary figures and images for: GB20-5A8-31, an anti-TL1A antibody for treating inflammatory bowel disease
Source: Front Immunol. 2026 Feb 3;17:1682346. doi: 10.3389/fimmu.2026.1682346 (PMC12911411; doi:10.3389/fimmu.2026.1682346)

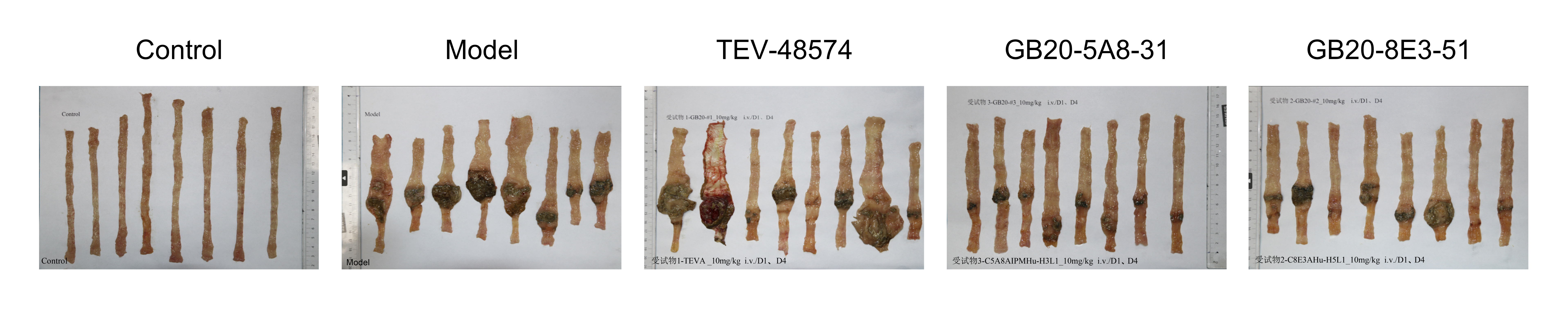

Supplement: Supplementary Figure 1 — The images of the colon at the endpoint of TNBS-induced rat IBD model. [file Image1.tif]

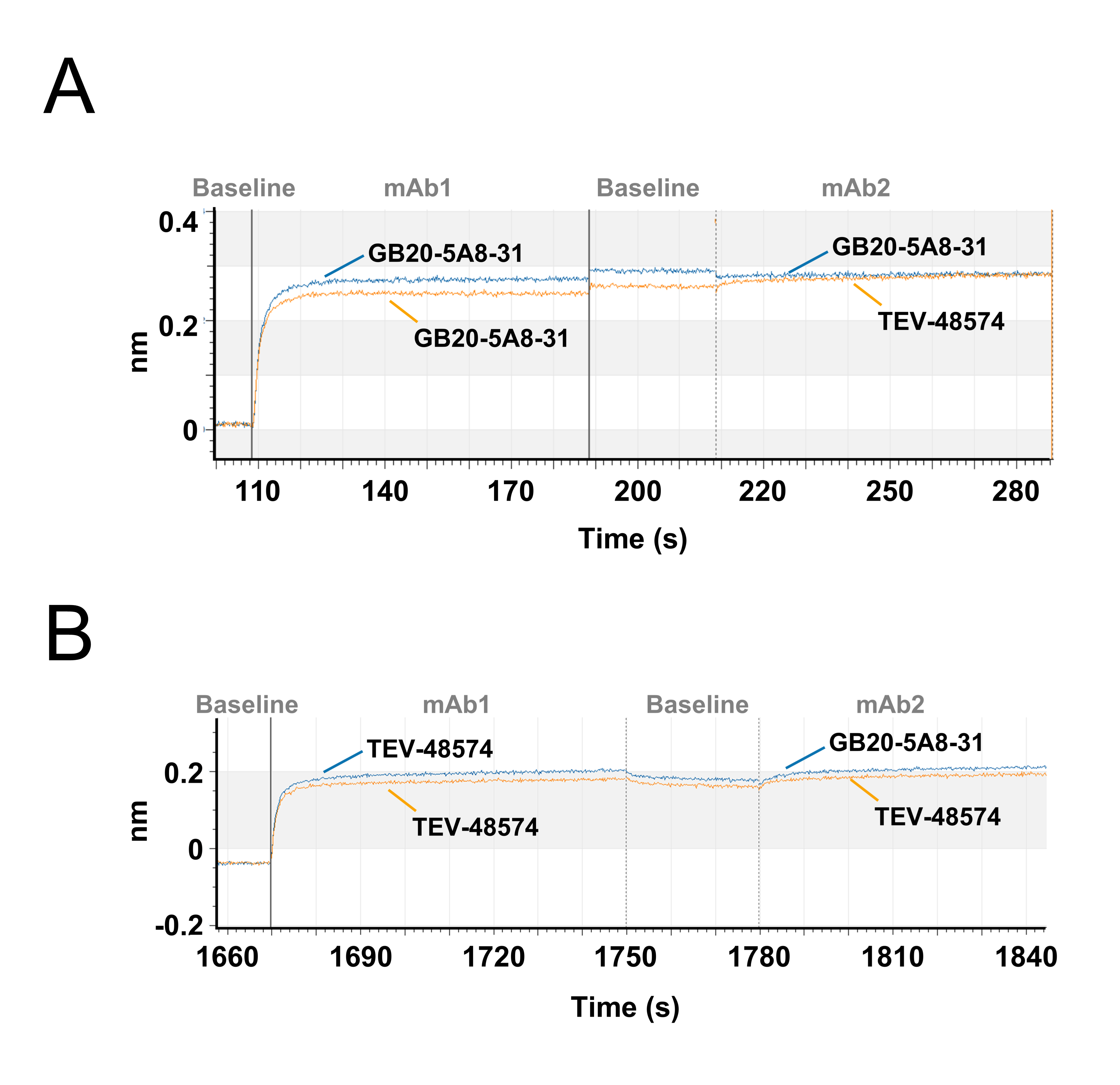

Supplement: Supplementary Figure 2 — Epitope binning analysis using Bio-Layer Interferometry (BLI). HIS1K biosensor tips were first loaded with 0.3 nM human TL1A, followed by sequential association with mAb1 and mAb2. (A) GB20-5A8–31 was applied as mAb1, subsequent association with either GB20-5A8–31 or TEV-48574 as mAb2. (B) TEV-48574 served as mAb1, the subsequent introduction of either GB20-5A8–31 or TEV-48574 as mAb2. [file Image2.tif]
