# Supplementary material for: GB20-5A8-31, an anti-TL1A antibody for treating inflammatory bowel disease
Source: Front Immunol. 2026 Feb 3;17:1682346. doi: 10.3389/fimmu.2026.1682346 (PMC12911411; doi:10.3389/fimmu.2026.1682346)
Supplement: Supplementary file 6 [file Table4.docx]

The biophysical properties of GB20-5A8-31 and its stability under stress conditions including freeze-thaw cycles (-80℃ for 24 h and 25℃ for 24 h, three times), elevated temperatures (40℃ for seven days), light exposure (4500lux ± 500lux, seven days), oxidation (1mM H_2_O_2_, 40℃, seven days), and pH variations (pH5.5 or 8.5, seven days) were characterized through the following analytical approaches.

Differential Scanning Fluorimetry

A differential scanning fluorimetry (DSF) assay was carried out using a Nano DSF system (Pronetheus NT48). The antibody sample was centrifuged at 12,000 × g for 10 min. Then, 20 μL of the supernatant was loaded into the capillaries, and the fluorescence signal was set within the range of 100 to 20,000. Following this, the temperature was raised from 20°C to 95°C at a rate of 1°C/min, while fluorescence signals were collected and analyzed.

Hydrophobic interaction chromatography assay

The antibody solubility index was assessed by hydrophobic interaction chromatography (HIC) using a Protemix HIC Butyl-NP5 column (4.6 × 100 mm, 5 μm) at 25°C. The mobile phase was 1.8 M ammonium sulfate in 0.1 M sodium phosphate (pH 6.5; A) and 0.1 M sodium phosphate (pH 6.5; B). The autosampler temperature was set at 8°C, the injection volume was 1 μg. A gradient elution was applied, linearly increasing mobile phase B from 44% to 100% over 20 min at a flow rate of 1.0 mL/min. The detection was carried out at 214 nm.

Size exclusion chromatography

The determination of antibodies’ purity was analyzed by the BioCore SEC-300 (NanoChrom, China) with PBS solution as mobile phase at a flow rate of 0.5 mL/min. The temperature of the column was set to 30 ℃. The acquisition time was set to 40 min. The samples were diluted with the mobile phase and centrifuged at 12,000 × g for 10 min at 4 °C. Finally, 50 μg of each sample was injected. The elution process was monitored by measuring the UV absorption at a wavelength of 280 nm. The purity of the purity of the sample is the percentage of the ratio of the main peak area to the total area.

Nonreducing capillary electrophoresis with sodium dodecyl sulfate

Capillary electrophoresis with sodium dodecyl sulfate under nonreducing condition (nrCE-SDS) was performed on Proteome Lab PA 800 plus system (AB Sciex, Redwood City, CA). In general, 40 μL of 2.5 mg/mL of sample and 5 μL of 0.25 M 2-Iodoacetamide and 55 μL of SDS sample buffer were mixed well, and centrifuge with a microcentrifuge to collect the liquid to the bottom of the tube. After incubated at 70 ℃ for 10 min, cooled at room temperature for a minimum of 3 min, vortexed, centrifuged at 6,000 × g for 1 min at 25 °C. 90 μL of supernatant was transferred into a micro sample tube. The effective separation length of capillary was 20 cm. Samples were introduced into the capillary at 5 kV for 20 s and separation by 15 kV for 40 min. UV detection of migrating proteins was monitored at 220 nm. The purity of the sample was indicated by the percentage of Time Corrected Area.

Bio-layer interferometry

The affinity between candidate Abs and hTL1A-trimer was determined using the Sartorius Octet® R8 Bio-layer Interferometry (BLI) system. The experiment was conducted in PBS at pH 7.4, supplemented with 0.05 % Tween 20, at a temperature of 25 °C. candidate or control molecule was immobilized onto ProA biosensors (Sartorius, #18-5010) until a BLI signal response of 1 nm was achieved. To measure association rates, the loaded biosensors were transferred to wells containing a 2-fold serially diluted hTL1A-His (Kactus Biosystems, #FSF-HM415), ranging from 0 to 100 nM. Dissociation rates were determined by dipping the biosensors into buffer-containing wells. The binding duration and dissociation time are adjusted depending on the analytes.
